# MyPath to Home Web-Based Application for the Geriatric Rehabilitation Program at Bruyère Continuing Care: User-Centered Design and Feasibility Testing Study

**DOI:** 10.2196/18169

**Published:** 2020-09-14

**Authors:** Chantal Backman, Anne Harley, Craig Kuziemsky, Jay Mercer, Liam Peyton

**Affiliations:** 1 School of Nursing Faculty of Health Sciences University of Ottawa Ottawa, ON Canada; 2 Bruyère Research Institute Ottawa, ON Canada; 3 Ottawa Hospital Research Institute Ottawa, ON Canada; 4 Bruyère Continuing Care Ottawa, ON Canada; 5 Faculty of Medicine University of Ottawa Ottawa, ON Canada; 6 MacEwan University Edmonton, AB Canada; 7 Faculty of Engineering University of Ottawa Ottawa, ON Canada

**Keywords:** geriatric rehabilitation, care transition, technology, hip fracture

## Abstract

**Background:**

When older adults return home from geriatric rehabilitation in a hospital, remembering the plethora of medical advice and medical instructions provided can be overwhelming for them and for their caregivers.

**Objective:**

The overall objective was to develop and test the feasibility of a novel web-based application called *MyPath to Home* that can be used to manage the personalized needs of geriatric rehabilitation patients during their transition from the hospital to home.

**Methods:**

This study involved (1) co-designing a patient- and clinician-tailored web-based application and (2) testing the feasibility of the application to manage the needs of geriatric rehabilitation patients when leaving the hospital. In phase 1, we followed a user-centered design process integrated with the modern agile software development methodology to iteratively co-design the application. The approach consisted of three cycles in which we engaged patients, caregivers, and clinicians to design a series of prototypes (cycles 1-3). In phase 2, we conducted a single-arm feasibility pilot test of *MyPath to Home*. Baseline and follow-up surveys, as well as select semistructured interviews were conducted.

**Results:**

In phase 1, semistructured interviews and talk-aloud sessions were conducted with patients/caregivers (n=5) and clinicians (n=17) to design the application. In phase 2, patients (n=30), caregivers (n=18), and clinicians (n=20) received access to use the application. Patients and their caregivers were asked to complete baseline and follow-up surveys. A total of 91% (21/23) of patients would recommend this application to other patients. In addition, clinicians (n=6) and patients/caregivers (n=6) were interviewed to obtain further details on the value of the web-based application with respect to engaging patients and facilitating communication and sharing of information with the health care team.

**Conclusions:**

We were successful at designing the *MyPath to Home* prototype for patients and their caregivers to engage with their clinicians during the transition from geriatric rehabilitation to home. Further work is needed to increase the uptake and usage by clinicians, and determine if this translates to meaningful changes in clinical and functional outcomes.

**International Registered Report Identifier (IRRID):**

RR2-10.2196/11031

## Introduction

Approximately 30,000 Canadians a year are admitted to the hospital with hip fractures [1]. With the increasingly aging population, our hospitals face constant pressure to discharge patients earlier, resulting in the need for more complex care to be delivered at home [2]. Best practice guidelines exist to ensure the quality of care of patients with hip fractures [3]. These guidelines recommend all patients with hip fractures receive active rehabilitation following their acute care stay, with rehabilitation beginning no later than 6 days following surgery [4]. This recommendation is consistent with findings that early access to an inpatient geriatric rehabilitation program after hip fracture increases the likelihood of patients returning home [5]. However, our challenge is that the health system does not always have the means to operationalize these guidelines.

When older adults return home from geriatric rehabilitation in a hospital, remembering all the medical advice and all the medical instructions can be overwhelming for them and for their caregivers. Care transition from the hospital to home is a vulnerable time, during which approximately one out of five adults experience an adverse event [6]. Communicating discharge instructions in an easy to understand way is very important [7]. Older adults and their caregivers require information that is accessible and can be easily shared with their primary care team. Currently, the paper discharge documents that patients receive are standardized, and we need to better accommodate the unique circumstances of each patient. The use of health information technologies can help patients and their caregivers access their personalized discharge information in order to better manage their health care needs while navigating our complex health care system [8]. The increased presence of mobile and wireless technologies, and advances in their application offer a potential solution to engage geriatric rehabilitation patients, engage them in shared decision making, and ultimately help them to better manage their personalized needs during care transition from the hospital to home. However, it is important that the intervention is well accepted, that it is used by the target population, and that it is adaptive to patients’ specific and evolving needs.

In order to improve the care transition from the hospital to home, hospitals need to improve how they engage patients and caregivers in terms of care, particularly during the discharge planning period [6]. Co-design is a participatory approach in which targeted end users, relevant clinicians, and researchers work together on all aspects of intervention development from needs assessment to content development, pilot testing, and dissemination [9,10]. This approach means that interventions are developed with an understanding of the local context and that the final product meets all stakeholder needs. Solutions designed in this way are more likely to be acceptable to both clinicians and patients/caregivers and therefore more likely to be adopted and sustained [11]. The purpose of this project was to co-design and test the feasibility of the novel *MyPath to Home* web-based application to manage the personalized needs of geriatric rehabilitation patients during their transition from the hospital to home.

## Methods

### Study Design and Setting

We conducted a user-centered design and feasibility testing study. The study took place in the Geriatric Rehabilitation Service at Bruyère Continuing Care, Ottawa, Ontario, Canada between July 2018 and March 2019. The study consisted of the following two phases: (1) co-designing the patient- and clinician-tailored *MyPath to Home* web-based application and (2) feasibility pilot testing of the application to manage the needs of geriatric rehabilitation patients when leaving the hospital. The detailed study protocol has been published elsewhere [12].

### Phase 1: Co-Design of the MyPath to Home Web-Based Application

#### Study Design

We followed a user-centered design process, integrated with a modern agile software development methodology [13-16]. The approach was iterative and consisted of three cycles in which we engaged patients, caregivers, and clinicians to design a series of prototypes of a patient- and clinician-tailored application (cycles 1-3), adjusting them according to end user feedback.

We collaborated with NexJ Health Inc, a provider of cloud-based population health management solutions, to design and configure the personalized care transition *MyPath to Home* web-based application (a minimum viable product for a mobile phone, tablet, or computer). The Connected Wellness platform of NexJ Health is a well-developed technology solution that is designed to support multichannel communications among patients, caregivers, and clinicians.

#### Participants

Posthip fracture surgery patients were eligible to participate based on the following inclusion criteria: (1) age 65 years or older; (2) English speaking; and (3) discharged to home or a community facility within the last 90 days. Caregivers aged 18 years or older who spoke English and any clinicians who were part of the Geriatric Rehabilitation Service were also eligible to participate. If a participant did not have their own personal mobile device or computer in the hospital, they were provided with a loaner iPad (Apple Inc). Additional training and support with the application were provided as needed.

#### Data Collection

##### Cycle 1: Modeling the Care Transition Process

In the modeling phase, we conducted a process mapping exercise and a needs assessment using semistructured interviews with patients, caregivers, and clinicians (physicians, nurses, physiotherapists, occupational therapists, and social workers) from the Geriatric Rehabilitation Service to identify the specific user requirements, workflow, goals, metrics, and data sources that would inform the design of the application. The different requirements were reconciled between the participant groups using a model to develop common ground regarding the system requirements [17].

##### Cycle 2: Implementation of the MyPath to Home Application

In the implementation cycle, the software development team and researchers (CB, LP, and CK) configured the *MyPath to Home* prototype and mapped the implementation of the clinical concepts and the inputs obtained from the intended users in the modeling phase.

##### Cycle 3: Evaluation of MyPath to Home Application Support for the Care Transition Process

In the evaluation cycle, we conducted audio-recorded talk-aloud sessions with the intended users (ie, patients, caregivers, and clinicians) to evaluate the usability of the personalized care transition *MyPath to Home* application while it was being used in real time. Participants were encouraged to talk aloud and provide feedback on the proposed step-by-step workflow and to comment on their experience with the prototype. Talk-aloud testing was chosen because “users verbalize their thoughts while performing prespecified tasks” [18]; this can be helpful in identifying potential barriers to adoption. Modifications to the software were based on user feedback in order to integrate patients’ and clinicians’ needs and preferences prior to the implementation of the single-arm feasibility pilot test.

#### Data Analysis

Interviews and talk-aloud sessions were recorded and transcribed verbatim. For the interview and talk-aloud transcripts, we conducted a qualitative content analysis [19] to provide a comprehensive and accurate descriptive summary of the participants’ perspectives. Two researchers (IG and CB) conducted the analysis independently. Data management software [20] was used to support the qualitative data analysis.

### Phase 2: Single-Arm Feasibility Pilot Test

#### Study Design

In phase 2, we conducted a single-arm feasibility pilot test of the *MyPath to Home* application. The specific objectives were to (1) determine whether it was feasible to provide the application to geriatric patients with hip fractures and their caregivers; (2) determine whether this application was acceptable to this population; and (3) refine the methods for a larger study.

#### Participants

We invited patients (n=30) who were being discharged from the Geriatric Rehabilitation Service to participate using a convenience sample. The sample size was not determined using sample size calculation, because the primary outcome of this study was not dependent on effect sizes. For feasibility studies, a sample size of approximately 24 to 50 has been previously recommended by other researchers [21-23]. The inclusion criteria were patients aged 65 years or older who had hip fracture surgery and informal caregivers aged 18 years or older who had access to a mobile or computer device.

#### Data Collection

##### Baseline (t=0)

After obtaining consent, patients, caregivers, and clinicians received training on how to use the *MyPath to Home* web-based application prior to obtaining access to it. Patients and their caregivers were asked to complete a survey of sociodemographic information (ie, age, gender, ethnicity, highest level of education, relationship status, and living situation) and the 16-item technology readiness index (TRI) 2.0, a scale that classifies participants by their level of technology adoption from 1.0 (low) to 5.0 (high) [24,25]. The TRI 2.0 has been verified for validity, reliability, and usefulness in a specified population subgroup like the one proposed in this study [26]. Patients and their caregivers were also asked to provide information about their specific needs and preferences (eg, goals of care), and review discharge and transition information (ie, workbooks). For the purpose of this study, a research assistant uploaded the discharge information from clinicians to the application.

##### Follow-up (t=1)

At 30 days after discharge, all patients (and their caregivers) were invited to complete a follow-up survey consisting of eight Likert scale questions. We also conducted select follow-up phone call interviews with patients (n=5), caregivers (n=1), and clinicians (n=6) to ask for their perspectives on the discharge processes and for their perspectives on the value of the *MyPath to Home* application with respect to its ability to engage patients and to facilitate communication and sharing of information with the health care team.

#### Data Analysis

We used descriptive statistics (ie, means) to summarize the survey results. The TRI 2.0 [24,25] was analyzed using mean scores for items that comprise the domains of *optimism*, *innovativeness*, *discomfort*, and *insecurity*. Scores were reverse coded for the inhibitor domains. A mean total score for technology readiness was computed.

Interviews were transcribed verbatim. The transcripts were analyzed independently by two researchers (IG and CB) using content analysis [19] to identify important contextual influences and practices related to the implementation and evaluation of the *MyPath to Home* web-based application.

## Results

### Demographics

Overall, 34 patients, 19 caregivers, and 20 clinicians participated in the study. The overall participant demographics for each study phase can be found in Table 1.

**Table 1:** Demographics of the study participants (N=73).

Occupation/role			Phase 1 Needs assessment, n		Phase 1 Talk-aloud sessions, n		Phase 2 Feasibility pilot, n
**Patients** (n=34)			1		3		30
	Male	0		1		11	
	Female	1		2		19	
Caregivers (n=19)			1		0		18
**Clinicians**^a^ (n=37)			6		11		20
	Physiotherapists	1		2		7	
	Social workers	1		2		3	
	Occupational therapists	1		2		4	
	Physicians	1		0		6	
	Nurses	1		4		0	
	Managers	1		1		0	

^a^A total of 20 unique clinicians participated in the study.

### Phase 1: Application Design

We developed the *MyPath to Home* web-based application to serve as a digital care transition record for geriatric patients with hip fractures. The *MyPath to Home* web-based application was designed to provide patients and their caregivers with access to all their personalized discharge information in one place. With the application, patients and their caregivers were able to securely access the discharge records and to access them seamlessly across a number of mobile devices, including smartphones, tablet computers, and laptop computers. The records were synchronized between these devices, helping the patients and their caregiver stay up to date.

According to the survey results, the *MyPath to Home* application had the key features requested by both patients/caregivers and clinicians. The five key features included (1) access to a discharge plan upon admission to geriatric rehabilitation; (2) sharing of preferences and needs with the “circle of care” team members; (3) access to multiple resources through the health library (ie, workbooks) on their dashboard; (4) access to their personal rehabilitation goals of care; and (5) access to personalized discharge information including discharge date, follow-up appointments, who to contact, equipment needs, home accommodation, community resources, and list of medications. Clinicians can review each of their patient’s specific preferences and needs during their rounds, assign specific resources to the health library (ie, workbooks), and upload all individualized discharge information and resources.

In addition, according to the information requirements identified in the study and with the help of clinicians, 11 workbooks were developed for patients and caregivers. These included general information about (1) geriatric rehabilitation; (2) equipment and devices (occupational therapy); (3) mobility (ie, list of physiotherapy community clinics, where to purchase gait aids, and medical supplies); (4) community resources; (5) safety in the home (preventing falls); (6) changes in behavior or mood; (7) safe medication use; (8) pain management; (9) when to call the doctor; (10) things to remember; and (11) additional web and telephone resources.

The talk-aloud transcripts were coded based on a usability testing framework [18]. Table 2 provides examples of participant suggestions for each of the domains of the framework (usability, visibility, content, understandability, usefulness navigation, and workflow).

**Table 2:** Examples of quotes.

Code	Examples
Usability	*I think you’ll have to try it to, to figure out what to modify. I do think a lot of them will struggle with the technology, but I think we should try it and see how it works and then make adaptations.* [Clinician #5]
Visibility	*So what’s missing is a phone number or a… an internet number of some kind.* [Patient #1] *So, this is their website.* [Interviewer]
Content	*Is there a self-care one? You have mobility, is there a self-care one there?* [Clinician #3]
Understandability	*I don’t understand the first question, which is when to call the doctor.* [Patient #1]
Usefulness	*Yes, this application is useful.* [Patient #1]
Navigation	*Go? Push this?* [Patient #2] *Yeah, you can press on the line.* [Interviewer]
Workflow	*Yeah. So if that’s something sort of at the start of their admission, then that, that would kind of take the burden off of them at the end.* [Clinician #3]

### Phase 2: Single-Arm Feasibility Pilot Test

#### Baseline (t=0)

In the baseline survey, we collected sociodemographic information on the participants (n=30). The mean age was 81 years. We had representation from both female participants (19/30, 63%) and male participants (11/30, 37%). Most patients lived alone (12/30, 40%) and were widowed (16/30, 53%). Further information on the participants can be found in Table 3.

**Table 3:** Sociodemographic information.

Patients		Value (N=30), n (%) or mean (range)	
Age (years)		81 (67-96)	
**Gender**			
	Female		19 (63%)
	Male		11 (37%)
**Ethnicity**			
	Caucasian		28 (93%)
	Other		2 (7%)
**Highest level of education completed**			
	Elementary school		4 (13%)
	High school		12 (40%)
	College level		5 (17%)
	University level		7 (23%)
	Graduate level		2 (7%)
**Relationship status**			
	Single/never married		2 (7%)
	Married or domestic partnership		11 (37%)
	Widowed		16 (53%)
	Separated		1 (3%)
**Living situation**			
	Alone		12 (40%)
	With partner		8 (27%)
	With children		3 (10%)
	With relatives		6 (20%)
	Retirement home		1 (3%)

The mean TRI 2.0 score was 3.26 out of a maximum score of 5, indicating a moderate level of technological adoption among the study cohort participants. The mean scores for innovativeness and optimism were 2.87 and 4.28, respectively. The mean scores for insecurity and discomfort were 2.57 and 3.32, respectively.

#### Usability of the Application (t=1)

During the pilot study period (phase 2), there were a total of 147 logins by patients, caregivers, and clinicians. Caregivers (n=16) accessed the application the most and also logged into the application the most frequently (38 logins). Usability data are provided in Table 4.

**Table 4:** Usability data.

Role	Value, n	logins 1-2, n	logins 3+, n	Total logins, n
Caregivers	16	11	5	38
Patients	7	3	4	26
Physicians	5	3	2	14
Physiotherapists	7	4	3	27
Occupational therapists	4	1	3	26
Social workers	3	1	2	16
Total	42	23	19	147

#### Follow-Up Surveys of Patients and Caregivers After Discharge (t=1)

In the 30-day postdischarge follow-up survey, patients and caregivers were questioned (n=23). Patients/caregivers felt that the information in the application was easy to understand (21/23, 91%), was helpful (21/23, 91%), helped to understand what they needed to do to prepare for discharge (22/23, 96%), and helped to identify the skills they needed to have for a successful discharge (20/23, 87%). Approximately 78% (18/23) of patients and caregivers found that the organization of the application made sense and that it was easy to navigate. Finally, 91% (21/23) of patients and caregivers would recommend this application to other patients.

#### Follow-Up Interviews With Patients, Caregivers, and Clinicians (t=1)

Patients (n=5), caregivers (n=1), and clinicians (n=6) participated in follow-up interviews. The clinicians who participated were physiotherapists (n=2), occupational therapists (n=2), a social worker (n=1), and a physician (n=1).

Participants described that an application, like *MyPath to Home*, was essential to help manage the personalized needs of geriatric rehabilitation patients during their transition from the hospital to home.

Specifically, patients and caregivers made the following statements:

This is the way to go!

Patient #2

Access to the application was quite helpful, the font was big enough!

Patient #4

Could be advertised more, some staff did not know about it.

Caregiver #1

Layout of the application and coaching on how to use it was good.

Patient #3

Good for someone who likes technology.

Caregiver #1

Having most of my care done at The Ottawa Hospital, this could be integrated with myChart.

Patient #2

Clinicians who used the application and participated in the follow-up interviews also described the challenges and benefits to this type of technology. Specifically, clinicians described the challenges with using this type of technology as follows:

This application added to my workload, it was more time consuming to log in.

Clinician #1

Easier use and having more mobile devices would be helpful.

Clinician #2

More education to the clinicians about the application would be useful.

Clinician #5

Despite these challenges, clinicians saw benefits of the technology. For example, “*It gave patients an opportunity to be involved in their care.*” [Clinician #4].

## Discussion

### Summary of the Findings

Our findings validate the use of *MyPath to Home* for geriatric rehabilitation patients after hip fracture, who are discharged from the hospital to home. This study directly integrated input and feedback from all relevant stakeholders (patients, caregivers, and clinicians) in the design and development of the personalized care transition application *MyPath to Home* for managing the needs of geriatric rehabilitation patients and for facilitating shared decision making. The application also included essential information as per the recommendations from Health Quality Ontario on patient care for patients with hip fractures and their caregivers [7].

Despite the co-design approach used in this study, there were still usability issues that emerged related to the clinician portion of this application. Further research on how this application can be better integrated into day-to-day practice is key to address the needs of all stakeholders involved [11]. These usability issues may have arisen owing to the nature of the research study, which was performed in addition to all regular day-to-day activities conducted by clinicians. Full integration of the application would require senior leadership support and a change in practice in order to be truly embedded into practice.

### Comparison With Previous Research

The use of web-based or mobile applications to assist patients during the transition between acute and subacute care is limited [27]. Our results were similar to those in another study [28], where barriers to the use of an application were identified. This included patient-related barriers and barriers related to the view of the application as being a second opinion and the application being seen as an external burden. As reported by Scott et al [28], an application needs to be highly engaging to improve uptake. In our study, we engaged clinicians, patients, and caregivers in the design of the web-based application in order to ensure adoption. The application was designed to provide tailored discharge and care transition information. Engaging patients and their caregivers in the discharge and care transition process is important to ensure that they are well prepared before going home, are active in their care, and are equal partners [29].

Our study was not designed to evaluate the impact of the application on health outcomes or care processes. However, we observed that some clinicians felt that the use of the application resulted in them having more control over care processes. We also observed that some patients felt they had more control during the care transition. However, there was no mention of substantial changes in their roles or in the relationship with clinicians. This may be because it was still too early to observe the effects on the patient-clinician relationship. However, it may also have to do with the limited integration with care processes or the limited information that can be accessed through the application. Furthermore, some patients and clinicians pointed out that the application played only a small role in their interactions.

The next step is to refine *MyPath to Home* and expand the application for the entire episode of care in the geriatric hip fracture population from admission to acute care, acute hospitalization, discharge from acute care, admission to geriatric rehabilitation, inpatient rehabilitation stay, and finally discharge to home or to the community.

This new and improved application will need to be further tested for effectiveness with a larger audience. Specifically, we will use the findings to inform a larger-scale study to develop an understanding of the specific mechanisms by which the *MyPath to Home* application is effective for patients, caregivers, and clinicians. We will test implementation and evaluate the technology-based intervention for effectiveness in a larger randomized study. Future research will more rigorously evaluate the health and economic benefits to inform wide-scale adoption of the technology.

### Strengths and Limitations

It is possible that the use of the technology led to unintended consequences (ie, increase in resource use rather than a decrease). For the purpose of this study, all data were entered directly into the application with no ability to receive data from other systems or send data to other systems; thus, some of the data might be duplicated in other systems (ie, hospital electronic health records). Future developments of this technology can include integration with electronic health records or other standard electronic health applications (ie, myChart).

### Conclusion

We were successful at designing the *MyPath to Home* prototype for patients and their caregivers to engage with their clinicians during the transition from geriatric rehabilitation to home. Further work is needed to increase the uptake and usage by clinicians, and determine if this translates to meaningful changes in clinical and functional outcomes. 

## Abbreviations

TRI: technology readiness index
